# THE IMPACT OF MOBILIZATION TRAINING TIME DURING THE FIRST POSTOPERATIVE WEEK ON THE LENGTH OF HOSPITAL STAY IN POSTOPERATIVE PATIENTS ADMITTED TO AN INTENSIVE CARE UNIT

**DOI:** 10.2340/jrm.v57.41015

**Published:** 2025-01-31

**Authors:** Yusuke OZAKI, Yuji KONO, Ayato SHINOHARA, Tomoyuki NAKAMURA, Takuma ISHIHARA, Osamu NISHIDA, Yohei OTAKA

**Affiliations:** 1 Department of Rehabilitation, Fujita Health University Hospital, Toyoake, Japan; 2 Department of Rehabilitation Medicine, School of Medicine, Fujita Health University, Toyoake, Japan; 3 Department of Anesthesiology and Critical Care Medicine, School of Medicine, Fujita Health University, Toyoake, Japan; 4 Innovative and Clinical Research Promotion Center, Gifu University Hospital, Gifu, Japan

**Keywords:** intensive care unit, postoperative period, postoperative care, rehabilitation, surgery

## Abstract

**Objective:**

To determine the impact of mobilization training time during the first postoperative week on the length of hospital stay for postoperative patients admitted to an intensive care unit.

**Design:**

A retrospective cohort study.

**Patients:**

Consecutive patients who underwent elective surgery and stayed in the intensive care unit of a university hospital for more than 48 h between July 2017 and August 2020 were enrolled.

**Methods:**

The total duration of mobilization training during the first postoperative week and clinical variables, including demographic information, were collected from medical records. Multivariable regression analysis was used to investigate the impact of mobilization training time on the length of hospital stay, adjusting for potentially confounding variables.

**Results:**

In total, 773 patients (504 males; median age, 70 years) were enrolled. Multivariable regression analysis showed that an increase in mobilization training time during the first postoperative week was associated with a shorter length of hospital stay (β = –0.067, 95% confidence interval: –0.120, –0.017, *p* = 0.010), with each 1-h increase in training time associated with a 4.02-day reduction in the length of hospital stay.

**Conclusion:**

Increased mobilization training during the first postoperative week significantly reduced the length of hospital stay in postoperative patients.

## LAY ABSTRACT

Prolonged postoperative bed rest poses risks such as muscle atrophy and limb weakness, particularly in patients in the intensive care unit. These conditions lead to difficulties in daily living after hospital discharge. Early mobilization, including sitting, standing, and walking, is important to counteract these risks; however, details regarding the effect of increasing the duration of early mobilization on reducing the length of hospital stay remain unclear. This study aimed to clarify whether mobilization time during the first postoperative week affected the length of hospital stay. Data from 773 patients admitted to the intensive care unit after elective surgery in a university hospital revealed that increasing the mobilization time by 1 h in the first postoperative week reduced the length of hospital stay by 4.02 days. This finding demonstrates the importance of early mobilization in reducing the length of hospital stay in patients admitted to the ICU after elective surgery.

The ageing of the population has substantially contributed to a growing proportion of patients being admitted to an intensive care unit (ICU). Approximately 5.7 million patients are admitted to the ICU each year, and the proportion of patients admitted after surgery is approximately 30% in the USA (1, 2). Prolonged postoperative bed rest leads to complications such as skeletal muscle atrophy, impaired exercise tolerance, and deep vein thrombosis (3, 4). Similarly, patients in the ICU are frequently prone to prolonged bed rest due to sedation, mechanical ventilation, and safety concerns regarding lines (5, 6) and spend more than 20 h per day on bed rest (7). A previous study reported that patients with acute respiratory failure or sepsis had a 19% decrease in the quadriceps muscle cross-sectional area 1 week after ICU admission (8). In addition, a prospective cohort study of patients who underwent coronary artery bypass grafting and were admitted to the ICU showed that prolonged bed rest increases the length of ICU stay, the duration of mechanical ventilation, and the length of hospital stay (9). Therefore, prolonged postoperative bed rest leads to complications and results in adverse outcomes for patients admitted to the ICU after surgery. Based on the aforementioned, it is necessary to consider effective countermeasures to prevent the various negative effects that can occur due to prolonged bed rest in patients admitted to the ICU after surgery.

The key countermeasure to shorten postoperative bed rest duration is early mobilization. Early mobilization is defined as physical activity that starts within 48–72 h after the onset of a critical illness or surgery (10, 11) and improves muscle strength and activities of daily living at hospital discharge in patients in the ICU (12, 13). Furthermore, early mobilization improves functional mobility at hospital discharge and decreases the length of ICU and hospital stays in surgical patients (2, 14). A high dose of early mobilization is associated with decreased 30-day mortality and improved functional capacity at ICU and hospital discharge (15), and early mobilization ≥ 40 min is associated with improved functional capacity of mobility and transfer and improved functional status at ICU discharge in patients in the ICU (16, 17). Therefore, early mobilization improves various physical functions and functional capacities; however, the exact impact of the early mobilization dose on the length of hospital stay, which is an important outcome for patients and has socioeconomic implications, has not been sufficiently investigated. Therefore, this study aimed to determine the impact of mobilization training time during the first postoperative week on the length of hospital stay of postoperative patients in the ICU.

## Methods

### Study design and setting

This retrospective cohort study was conducted at the Fujita Health University Hospital in Aichi, Japan. The Research Ethics Committee of Fujita Health University approved the study protocol (Approval No: HM22–051), and the results were reported in accordance with the STROBE guidelines.

### Participants

From July 2017 to August 2020, consecutive patients who underwent elective surgery and were admitted to the ICU at Fujita Health University Hospital for more than 48 h were enrolled. Patients were excluded if they were: (*i*) younger than 18 years old, (*ii*) could not go out using public transport or could not go out in the neighbourhood before surgery (18), (*iii*) had paralysis, (*iv*) had cerebrovascular diseases, (v) had amputations in the lower extremity, and (*vi*) died in the ICU. The requirement for informed consent was waived owing to the retrospective study design, and individuals who did not opt out were included.

### Data collection

Demographic and clinical information, including age, sex, body mass index, acute physiology and chronic health evaluation II score (APACHE II score), Charlson comorbidity index (19), disease name, type of operation, operation time, duration of anaesthesia, total blood loss, postoperative complications (pulmonary complications, anastomotic leakage, surgical site infection, postoperative haemorrhage, and acute kidney injury), length of ICU stay, length of mechanical ventilation, and length of hospital stay were collected from patients’ medical records. The Medical Research Council (MRC) and functional status scores for the ICU (FSS-ICU) at ICU discharge (20) were collected as measures of muscle strength and functional status, respectively. Information on rehabilitation was collected, including the starting timing of sitting on the edge of the bed, standing, walking, and the timing when independent walking was achieved. The total time for rehabilitation sessions and mobilization training during the first postoperative week was also collected. In this study, mobilization training was defined as sitting on the edge of the bed, standing, and walking (21). Mobilization training and total rehabilitation time during the first postoperative week were recorded in 5-min increments.

### Rehabilitation sessions

Rehabilitation in the ICU was provided 7 days a week by experienced physical therapists based on a standard protocol. The contents of rehabilitation sessions in the ICU consisted of mobilization training, muscle strengthening exercises, range of motion exercises, and postural drainage. After discharge from the ICU, rehabilitation was provided 5 days a week by a physical therapist or an occupational therapist according to the patient’s condition. The contents of the rehabilitation sessions after discharge from the ICU consisted of muscle-strengthening exercises, sitting on the edge of the bed, standing, walking, balancing exercises, and stair exercises depending on the patient’s condition.

### Statistical analysis

Patient characteristics are presented as medians and interquartile ranges for continuous variables and percentages for categorical variables. A multivariable linear regression model was used to confirm the effect of mobilization training time during the first postoperative week on the length of hospital stay (Model 1). We also used multivariable linear regression analysis to assess the effect of the total training time during the first postoperative week on the length of hospital stay (Model 2). Furthermore, because the rehabilitation training time was not the same among patients, a regression model was constructed for the mobilization training time percentages (mobilization training time/rehabilitation total time) (Model 3). Possible confounding factors were considered from clinical and statistical perspectives and included as covariates in the multivariable regression model. All analyses were performed using JMP13 software (SAS Institute Inc, Cary, NC, USA), and the two-sided significance level was set at 0.05.

## Results

**Fig. 1:**
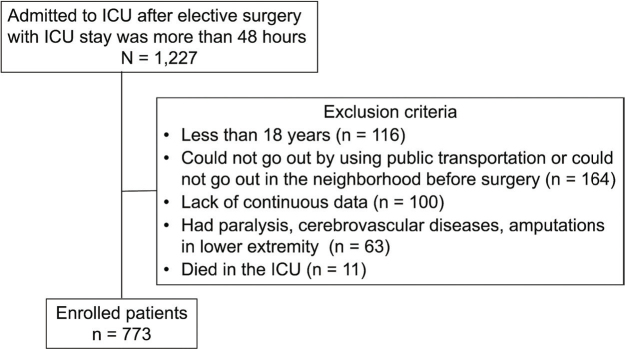
Flow of participants.

**Fig. 2:**
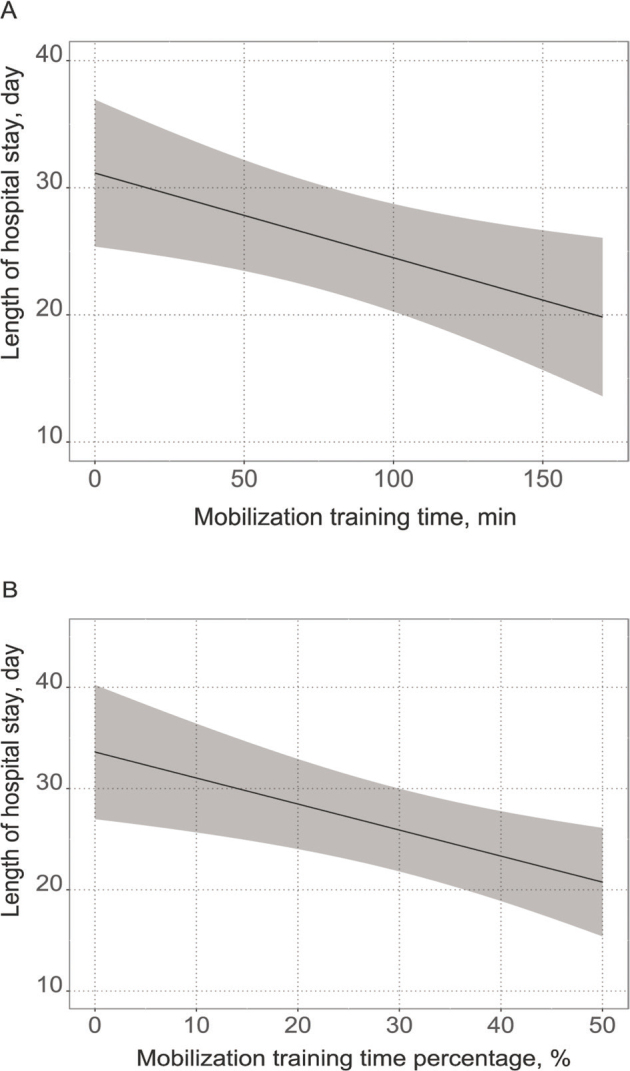
Association between length of hospital stay and mobilization training time. Possible confounding factors were included as covariates in the multivariable regression models. In these figures, values of covariates are fixed at their medians. (A) Association between length of hospital stay and mobilization training time using a multivariable linear regression model. (B) Association between length of hospital stay and mobilization training time percentage (mobilization training time/rehabilitation total time) using a multivariable linear regression model.

The participant flow is shown in Fig. 1. Among the 1,227 enrolled patients, 773 (504 males, median age, 70 years) were included in the analysis. The patients’ clinical characteristics are presented in Table I (details of training time are given in Table SI). The most common type of surgery was cardiovascular (47.5%), followed by gastrointestinal (23.4%). The participants had relatively mild comorbidities with an APACHE II score of 14.0 (11.0–16.0) and a Charlson comorbidity index score of 0 (0–0). The mobilization training time during the first postoperative week was 70.0 min (40.0–95.0), and the mobilization training time percentage during the first postoperative week was 26.9% (20.0–29.7). The results of the univariable regression analyses are presented in Table II. Body mass index, APACHE II score, shortening the starting timing of walking, and increasing mobilization training time during the first postoperative week were associated with a decreased length of hospital stay, whereas an increased total rehabilitation time during the first postoperative week was associated with an increased length of hospital stay. The results of the multivariable regression analyses (models 1–3) are presented in Table III. Detailed statistics, including covariates, are shown in Tables SII–SIV. The multivariable regression analyses (model 1 and model 3) showed that the length of hospital stay was associated with mobilization training time (β = –0.067, *p* = 0.010) and mobilization training time percentage (β = –0.260, *p* = 0.003) during the first postoperative week. Increasing the mobilization time by 1 h during the first postoperative week shortened the length of hospital stay by 4.02 days, and increasing the mobilization time percentage by 10% during the first postoperative week shortened the length of hospital stay by 2.6 days. However, the total rehabilitation time during the first postoperative week, which was associated with the length of hospital stay in the univariable regression analysis, was no longer associated with the length of hospital stay in the multivariable analysis. The predicted values obtained from the multivariable regression models for the length of hospital stay relative to the mobilization training time and the mobilization training time percentage are shown in Fig. 2.

**Table I:** Clinical characteristics (*n* = 773)

Variables	All patients (*n* = 773)
Age, years, median (IQR)	70.0 (61.0–77.0)
Sex, male, *n* (%)	269 (34.8%)
APACHE II score, median (IQR)	14.0 (11.0–16.0)
Charlson comorbidity index score, median (IQR)	0 (0–0)
Body mass index, kg/m^2^, median (IQR)	22.7 (20.1–25.2)
Normal, *n* (%)	472 (61.1)
Underweight, *n* (%)	87 (11.3)
Overweight, *n* (%)	174 (22.5)
Obese, *n* (%)	40 (5.2)
Disease, *n* (%)	
Cardiovascular disease	367 (47.5)
Cancer	301 (38.9)
Other	105 (13.6)
Type of operation, *n* (%)	
Cardiovascular surgery	367 (47.5)
Gastrointestinal surgery	181 (23.4)
Thoracic surgery	83 (10.7)
Other	142 (18.4)
Operation information	
Operation time, min, median (IQR)	410 (308–631)
Duration of anaesthesia, min, median (IQR)	511 (395–737)
Total blood loss, mL, median (IQR)	412 (184–732)
Postoperative complications, *n* (%)	
Postoperative pulmonary complications	141 (18.2)
Anastomotic leakage, surgical site infection, postoperative haemorrhage	4 (0.5)
Acute kidney injury	4 (0.5)
Physical functions, median (IQR)	
MRC sum score at ICU discharge	60 (58–60)
FSS-ICU sum score at ICU discharge	16 (12–18)
Rehabilitation progress and dose, median (IQR)	
Length of ICU stay, days	4.0 (3.0–5.0)
Length of mechanical ventilation, days	2.0 (1.0–2.0)
Length of hospital stay, days	23.0 (17.0–37.0)
Starting timing of sitting on the edge of the bed, day	2.0 (2.0–3.0)
Starting timing of standing, day	3.0 (2.0–4.0)
Starting timing of walking, day	6.0 (5.0–8.0)
Timing when independent walking was acquired, day	9.0 (7.0–15.0)
Rehabilitation total time during the first postoperative week, min	260.0 (200.0–320.0)
Mobilization training time during the first postoperative week, min	70.0 (40.0–95.0)
Mobilization training time percentage during the first postoperative week, %	26.9 (20.0–29.7)
Discharge place, *n* (%)	
Home	727 (94.0)
Hospital or facilities	33 (4.3)
Death	13 (1.7)

APACHE II: Acute Physiology and Chronic Health Evaluation II; ICU: intensive care unit; MRC: Medical Research Council; FSS-ICU: functional status scores for the ICU.

**Table II:** Univariable regression analyses for length of hospital stay

Variables	β	95% CI	*p*-value
Rehabilitation progress and dose			
Rehabilitation total time during the first postoperative week, min	0.036	0.015, 0.060	0.003
Mobilization training time during the first postoperative week, min	–0.156	–0.208, –0.104	<0.001
Mobilization training time percentage during the first postoperative week, %	–0.664	–0.829, –0.498	<0.001
Starting timing of sitting on the edge of the bed, day	3.511	2.444, 4.579	<0.001
Starting timing of standing, day	3.497	2.806, 4.189	<0.001
Starting timing of walking, day	1.978	1.635, 2.321	<0.001
Timing when independent walking was acquired, day	0.906	0.791, 1.020	<0.001
Length of ICU stay, days	0.433	–0.205, 1.072	0.183
Length of mechanical ventilation, days	2.116	1.256, 2.975	<0.001
Age, years	–0.018	–0.185, 0.150	0.837
Sex, male, *n*	0.264	–1.969, 2.497	0.816
APACHE II score	0.856	0.296, 1.416	0.003
Charlson comorbidity index score	0.780	–1.681, 3.241	0.534
Body mass index, kg/m^2^		–	
Normal (ref), *n*	–	–	–
Underweight, *n*	6.551	1.141, 11.961	0.018
Overweight, *n*	–6.521	–10.925, –2.117	0.004
Obese, *n*	1.561	–5.682, 8.805	0.672
Disease			
Cardiovascular disease (ref), *n*	–	–	–
Cancer, *n*	4.704	1.651, 7.756	0.003
Other, *n*	5.466	1.453, 9.480	0.008
Type of operation			
Cardiovascular surgery (ref), *n*	–	–	–
Gastrointestinal surgery, *n*	0.149	–3.668, 3.967	0.939
Thoracic surgery, *n*	2.709	–2.312, 7.729	0.290
Other, *n*	8.589	4.461, 12.718	<0.001
Operation information			
Operation time, h	0.017	0.009, 0.026	<0.001
Duration of anaesthesia, h	0.015	0.007, 0.023	<0.001
Total blood loss, mL	0.001	–0.001, 0.004	0.201
Postoperative complications			
Pulmonary complications, *n*	–1.865	–4.616, 0.886	0.184
Anastomotic leakage, surgical site infection, postoperative haemorrhage, *n*	19.543	4.783, 34.304	0.010
Acute kidney injury, *n*	30.035	15.363, 44.0	<0.001
Physical function			
MRC sum score at ICU discharge	–0.517	–1.011, –0.022	0.041
FSS-ICU sum score at ICU discharge	–1.013	–1.396, –0.630	<0.001

CI: confidence interval; APACHE II: Acute Physiology and Chronic Health Evaluation II; ICU: intensive care unit; MRC: Medical Research Council; FSS-ICU: functional status scores for the ICU.

**Table III:** Multivariable regression analyses for length of hospital stay

Model	Variable	Β	95% CI	*p*-value
1	Mobilization training time, min	–0.067	–0.120, –0.017	0.010
2	Rehabilitation total time, min	0.015	–0.008, 0.038	0.197
3	Mobilization training time, %	–0.260	–0.433, –0.087	0.003

All multivariable regression analyses (models 1–3) were adjusted for demographic and clinical information (age, sex, body mass index, Acute Physiology and Chronic Health Evaluation II score, Charlson comorbidity index, and type of operation), operation information (operation time and total blood loss), postoperative complications (pulmonary complications, anastomotic leakage, surgical site infection, postoperative haemorrhage, and acute kidney injury), starting timing of walking, length of intensive care unit stay, and length of mechanical ventilation.

CI: confidence interval.

## DISCUSSION

This is the first large-scale observational study to clarify the impact of the mobilization dose on the length of hospital stay during the early postoperative period in patients admitted to the ICU. The mobilization training time during the first postoperative week was associated with the length of hospital stay, with each 1 h increase in training time associated with a 4.02-day reduction.

A previous study on early mobilization dose reported that early mobilization ≥ 40 min daily improved the functional capacity of patients at ICU discharge more than those mobilized for < 40 min daily (16). Furthermore, in patients in the ICU who were mechanically ventilated for more than 24 h, early mobilization for 19 min per day during mechanical ventilation improved functional capacity at discharge compared with patients who were not mobilized during mechanical ventilation (22). Although the median mobilization training time during the postoperative week was 70 min in the present study, which was shorter than the mobilization dose in the previous study, the findings were consistent, with a decreased length of hospital stay as the mobilization time increased. Two possible reasons exist for the difference in dose between our study and the previous study. First, a previous study on early mobilization included rehabilitation sessions and those outside the rehabilitation session (16); another study included mobilization and passive range of motion exercises (22). In contrast, the present study included only mobilization training during rehabilitation sessions. Second, the previous studies (16, 22) included postoperative patients in the ICU and neurocritical and medical patients in the ICU. In contrast, the present study included only postoperative patients admitted to the ICU who underwent elective surgery. The present study adds to the knowledge that increasing mobilization training time was associated with a decreased hospital stay in patients with mild comorbidities in the postoperative ICU, even with a relatively short mobilization training time. Interestingly, in contrast to our findings and those of previous studies (16, 22), it was reported that a high dose of early mobilization consisting of 3 sessions per day did not shorten the length of hospital stay compared with usual care in patients with acute respiratory failure admitted to the ICU (23). Furthermore, early mobilization of 30–60 min/day did not shorten the length of hospital stay compared with early mobilization of 5–10 min/day in medical patients in the ICU (24). The results of previous studies (16, 22–24) and ours indicate that the dose effects of mobilization training on patients’ conditions and the range of doses are not well understood. Future studies should explore the type of patients and how the dose is administered.

Interestingly, a positive correlation was observed between total rehabilitation time during the first postoperative week and length of hospital stay in the univariable regression analysis. This may be because the total rehabilitation time was longer in severely ill patients who were in high need of rehabilitation. However, the multivariable linear regression analysis adjusted for variables, including illness severity, revealed no association between total rehabilitation time and length of hospital stay. Furthermore, to adjust for differences in the total rehabilitation time among the participants, we applied the percentage of mobilization training time instead of the mobilization training time in another multivariable regression analysis, which confirmed that a larger percentage of mobilization training was associated with a shorter hospital stay.

Considering the findings that increasing mobilization training time during the first postoperative week had a significant impact on the length of hospital stay, even in postoperative patients admitted to the ICU with short mobilization training time, this study emphasizes the importance of increasing mobilization training time during the early postoperative period, even in patients with relatively mild comorbidities in the postoperative ICU. Therefore, this study showed that, in postoperative patients admitted to the ICU who underwent elective surgery, it is more important to increase the mobilization training time and mobilization time percentage as much as possible, including outside of rehabilitation, rather than increasing the rehabilitation time during the first postoperative week to shorten the length of hospital stay.

This study has some potential limitations. First, patients were recruited from a single-centre registry. Therefore, the results may not be generalizable to groups with dissimilar demographic characteristics. Furthermore, the results of mobilization training time during the first postoperative week excluded mobilization time outside of rehabilitation. Previous studies have reported that the length of hospital stay increased in postoperative gastrointestinal surgery patients with decreased frequency of postoperative walking (25). In the present study, patients stood up on average on the 3^rd^ postoperative day and began walking on the 6^th^ postoperative day. Patients were also considered to have been using a wheelchair and walking outside of rehabilitation during the first postoperative week, and the time of mobilization, including sitting and walking outside of rehabilitation, may be related to the length of hospital stay. Therefore, in the future, it will be necessary to clarify the relationship between the total amount of activity, including mobilization time outside of rehabilitation, and the length of hospital stay in postoperative patients admitted to the ICU who have undergone elective surgery. Nevertheless, the results of this study provided new findings that mobilization training time during the early postoperative period could be associated with the length of hospital stay for postoperative patients admitted to the ICU.

In conclusion, this study showed that mobilization training time during the first postoperative week is an independent factor associated with the length of hospital stay and significantly impacts the length of hospital stay in postoperative patients in the ICU who undergo elective surgery.

## Supplementary Material

THE IMPACT OF MOBILIZATION TRAINING TIME DURING THE FIRST POSTOPERATIVE WEEK ON THE LENGTH OF HOSPITAL STAY IN POSTOPERATIVE PATIENTS ADMITTED TO AN INTENSIVE CARE UNIT
