# Supplementary material for: THE IMPACT OF MOBILIZATION TRAINING TIME DURING THE FIRST POSTOPERATIVE WEEK ON THE LENGTH OF HOSPITAL STAY IN POSTOPERATIVE PATIENTS ADMITTED TO AN INTENSIVE CARE UNIT
Source: J Rehabil Med. 2025 Jan 31;57:41015. doi: 10.2340/jrm.v57.41015 (PMC11811531; doi:10.2340/jrm.v57.41015)
Supplement: THE IMPACT OF MOBILIZATION TRAINING TIME DURING THE FIRST POSTOPERATIVE WEEK ON THE LENGTH OF HOSPITAL STAY IN POSTOPERATIVE PATIENTS ADMITTED TO AN INTENSIVE CARE UNIT [file JRM-57-41015-s1.pdf]

Supplementary material has been published as submitted. It has not been copyedited, or typeset by Journal of Rehabilitation Medicine

Table SI. Details of mobilization training time in the first postoperative week

Variables	All patients (n = 773)	
Mobilization training time per day, min	18.3	(14.0–22.5)
Percentage of sitting on the edge of the bed in mobilization training time, %	45.5	(27.3–61.5)
Percentage of standing in mobilization training time, %	20.0	(8.3–32.0)
Percentage of walking in mobilization training time, %	22.7	(0–45.0)

Data are presented as median (interquartile range).

Table SII. Multivariable linear regression analysis to confirm the association between mobilization training time and the length of hospital stay

Variables	β	95% CI	p-value
Mobilization training time during the first postoperative week, min	-0.067	-0.120, -0.017	0.010
Age, years	0.186	0.011, 0.334	0.037
Sex, male, n	0.712	-1.625, 2.455	0.690
APACHEII score	0.273	-0.289, 0.791	0.362
Charlson comorbidity index score	0.490	-1.982, 2.892	0.714
Body mass index, kg/m^2			
Normal (ref), n	-	-	-
Underweight, n	4.739	-0.258, 9.736	0.063
Overweight, n	-6.482	-10.516, -2.448	0.017
Obese, n	3.788	-2.943, 10.519	0.270
Type of operation			
Cardiovascular surgery (ref), n	-	-	-
Gastrointestinal surgery, n	-0.984	-4.726, 2.758	0.606
Thoracic surgery, n	2.015	-2.849, 6.879	0.416
Other, n	6.972	2.834, 11.111	0.001
Operation information			
Operation time, hour	0.009	0.003, 0.018	0.043
Total blood loss, 100mL	-0.098	-0.310, 0.118	0.376
Postoperative complications			
Pulmonary complications, n	-0.767	-3.635, 2.10	0.599
Anastomotic leakage, surgical site infection, postoperative haemorrhage, n	20.321	6.996, 33.646	0.003
Acute kidney injury, n	13.105	-0.615, 26.824	0.061
Rehabilitation progress and dose			
Starting timing of walking, day	1.537	1.161, 1.914	<0.001
Length of ICU stay, day	0.139	-0.435, 0.713	0.635
Length of mechanical ventilation, day	0.266	-0.573, 1.105	0.544

CI, confidence interval; APACHE II, Acute Physiology and Chronic Health Evaluation II; ICU, intensive care unit

Table SIII. Multivariable linear regression analysis to confirm the association between rehabilitation total time and the length of hospital stay

Variables	β	95%CI	p-value
Rehabilitation total time during the first postoperative week, min	0.015	-0.008, 0.038	0.197
Age, years	0.169	0.007, 0.332	0.041
Sex, male, n	0.872	-1.178, 2.921	0.404
APACHEII score	0.230	0.312, 0.772	0.406
Charlson comorbidity index score	0.714	-1.726, 3.153	0.566
Body mass index, kg/m^2			
Normal (ref), n	-	-	-
Underweight, n	4.997	-0.016, 10.009	0.051
Overweight, n	-6.707	-10.752, -2.661	0.012
Obese, n	3.945	-2.807, 10.697	0.252
Type of operation			
Cardiovascular surgery (ref), n	-	-	-
Gastrointestinal surgery, n	-1.207	-4.963, 2.550	0.529
Thoracic surgery, n	1.235	-3.667, 6.137	0.621
Other surgery, n	8.464	4.299, 12.628	<0.001
Operation information			
Operation time, hour	0.010	0.001, 0.020	0.024
Total blood loss, 100mL	-0.084	-0.301, 0.133	0.446
Postoperative complications			
Pulmonary complications, n	-1.133	-4.015, 1.748	0.440
Anastomotic leakage, surgical site infection, postoperative haemorrhage, n	20.695	7.329, 34.060	0.003
Acute kidney injury, n	13.288	-0.480, 27.056	0.059
Rehabilitation progress and dose			
Starting timing of walking, day	1.578	1.201, 1.956	<0.001
Length of ICU stay, day	0.20	-0.376, 0.776	0.496
Length of mechanical ventilation, day	0.258	-0.589, 1.105	0.550

CI, confidence interval; APACHE II, Acute Physiology and Chronic Health Evaluation II; ICU, intensive care unit

Table SIV. Multivariable linear regression analysis to confirm the association between mobilization training time percentage and the length of hospital stay

Variables	β	95%CI	p-value
Mobilization training time percentage during the first postoperative week, %	-0.260	-0.433, -0.087	0.003
Age, years	0.181	0.019, 0.342	0.028
Sex, male, n	0.835	-1.20, 2.871	0.421
APACHEII score	0.251	-0.287, 0.789	0.359
Charlson comorbidity index score	0.496	-1.930, 2.923	0.688
Body mass index, kg/m^2			
Normal (ref), n	-	-	-
Underweight, n	4.498	-0.498, 9.494	0.078
Overweight, n	-6.346	-10.377, -2.315	0.002
Obese, n	3.781	-2.939, 10.502	0.270
Type of operation			
Cardiovascular surgery (ref), n	-	-	-
Gastrointestinal surgery, n	-1.113	-4.848, 2.622	-0.559
Thoracic surgery, n	1.636	-3.210, 6.482	0.508
Other surgery, n	7.318	3.232, 11.403	<0.001
Operation information			
Operation time, hour	0.009	-0.001, 0.018	0.054
Total blood loss, 100mL	-0.098	-0.314, 0.117	0.372
Postoperative complications			
Pulmonary complications, n	-0.887	-3.746, 1.972	0.543
Anastomotic leakage, surgical site infection, postoperative haemorrhage, n	20.136	6.829, 33.443	0.003
Acute kidney injury, n	13.512	-0.189, 27.213	0.053
Rehabilitation progress and dose			
Starting timing of walking, day	1.457	1.071, 1.843	<0.001
Length of ICU stay, day	0.138	-0.435, 0.711	0.638
Length of mechanical ventilation, day	0.183	-0.660, 1.025	0.670

CI, confidence interval; APACHE II, Acute Physiology and Chronic Health Evaluation II; ICU, intensive care unit
